# Two cases of pancreatic tuberculosis in immunocompetent individuals presenting as diabetes mellitus: An overview of clinical features, diagnosis and management

**DOI:** 10.5339/qmj.2024.54

**Published:** 2024-10-07

**Authors:** G Varadaraj, Avantika Rai, Parthasarathi Ghana, Kiran Maribashetti

**Affiliations:** 1 Army College of Medical Sciences, New Delhi, India *Email: flt.lt.varad@gmail.com; 2 12 Air Force Hospital, Gorakhpur, India; 3 Air Force Central Medical Establishment, New Delhi, India; 4 155 Base Hospital, Tezpur, India

**Keywords:** Pancreatic diabetes, overview TB pancreas, pancreatic tuberculosis

## Abstract

Mycobacterium tuberculosis (TB) disease is a major global health problem affecting 10.6 million people worldwide, and India alone contributes 28% to this burden. Pancreatic TB is considered an extremely rare entity which closely mimics pancreatic carcinoma. However, while 87% of the total globally notified TB cases are from 30 high-burden countries mostly from Asia, about 45% of pancreatic TB cases are from developed countries. This suggests that the identified cases of pancreatic TB in developing counties are just the tip of iceberg, and many cases either remain undetected or wrongly diagnosed. The majority of the patients with pancreatic TB undergo extensive evaluation and frequently undergo major abdominal surgeries since pancreatic carcinoma is often considered as the most probable diagnosis before TB. Here, 2 cases of pancreatic TB managed separately at two different centers are described. Both cases presented with significant unintentional weight loss and poor glycemic control which is an unusual presentation for pancreatic TB. Imaging showed pancreatic mass lesions, and initially pancreatic carcinoma was considered by the treating physicians. Chromogranin A in both patients and tumor markers done in one of the patients were negative. Both the patients underwent Ga-68 DOTANOC PET/CT scan, and one of the patients even showed multiple DOTANOC avid lesions. Owing to contradictory biochemical and imaging findings, biopsy of the pancreatic mass was done which clinched the diagnosis of pancreatic TB. On starting standard anti-tubercular therapy (ATT), both patients showed clinical and radiological recovery with a significant regain of glycemic control. The clinical features, appropriate investigation including imaging and tissue biopsy, and treatment options are described in the article for better understanding of the disease. This may guide clinicians in early detection of pancreatic TB with least invasive diagnostic procedures.

## 1. Introduction

Mycobacterium tuberculosis (TB) remains a significant cause of morbidity and mortality despite advancement in the medical field, affecting all groups of people without any age or gender predilection.^1^ Despite the concentrated efforts by the World Health Organization (WHO), TB continues to remain as the leading cause of death from a single infection agent, affecting 10.6 million worldwide, and India alone contributes nearly a quarter to this burden.^1,2^

Pulmonary TB constitutes to the majority of TB cases, and pancreatic TB is considered an extremely rare entity which closely mimics pancreatic carcinoma.^3^ The majority of the patients with pancreatic TB undergo extensive evaluation and frequently undergo major abdominal surgeries since pancreatic carcinoma is often considered as the most probable diagnosis before TB.^3,4^ The main objective of this article is not to showcase how ideally the 2 cases were managed but to highlight the treatment dilemmas and pitfalls in managing them.

## 2. Case Presentation

### 2.1. Case 1

A 26-year-old lady presented to medical out-patient-department (OPD) OPD with a history of significant unintentional weight loss of 14 kg over the past 2 months associated with excessive thirst (polydipsia) and palpitations. On further questioning, she admitted to having vague abdominal pain for the past 2–3 months. There was no history of fever, cough, jaundice, or diarrhoea. On evaluation, she was hemodynamically stable; general and systemic examination were unremarkable. On investigation, complete blood count (CBC), liver function tests (Bilirubin, AST, and ALT), and renal function tests (urea and creatinine) were within normal limits. However, her fasting plasma glucose (FPG) was highly elevated (427 mg/dL), and HbA1C was 10.1%. Her thyroid function tests [Free T3, Free T4, and ultrasensitive thyroid stimulating hormone (TSH)] were found to be within normal limits. Screening for human immunodeficiency virus (HIV) was negative. Also, serum sodium, potassium, calcium, and parathormone levels were within normal range. Patient was started on basal-bolus insulin for control of blood glucose levels.

**Figure 1.**
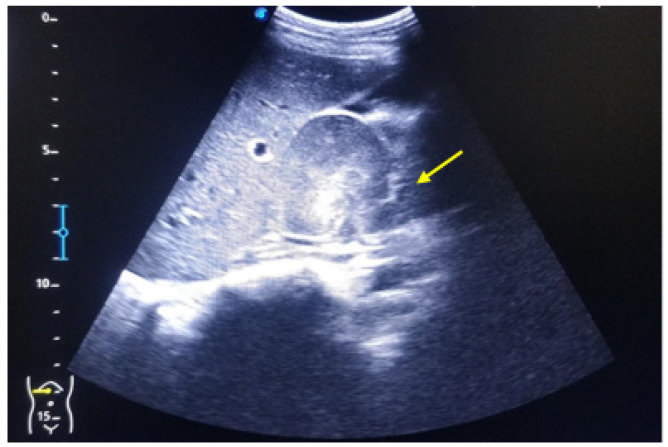
USG showing right-sided para-renal mass impinging on liver.

**Figure 2.**
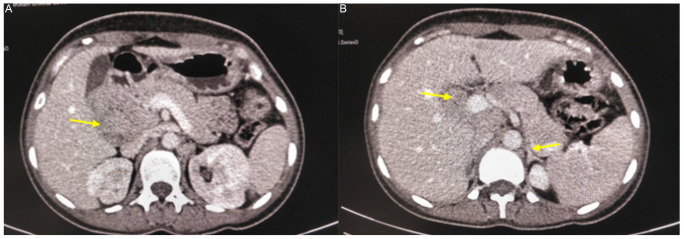
(A) CECT showing homogenously enhancing pancreatic mass. (B) CECT showing pancreatic mass abutting duodenum and adrenal gland.

Abdominal Ultrasound (USG) showed right-sided para-renal mass (Figure 1). Hence, contrast-enhanced computed tomography (CECT) Abdomen was done which revealed a homogenously enhancing mass lesion in the pancreatico-duodenal groove measuring 46×50×58 mm which showed post-contrast enhancement with non-enhancing area within (Figure 2A). Pancreatic head and uncinated process, fat plane between duodenum and the mass lesion, were not clearly visualized in CECT Abdomen (Figure 2B). Also, the pancreatico-duodenal groove was widened, and the D2-D3 segment of duodenum was displaced toward right lateral aspect. In the postero-superior aspect, the lesion was not seen separate from the confluence of right adrenal gland and inferior vena cava (IVC). There were multiple nodular enhancing lesions in conglomeration suggestive of lymph nodes in the aortocaval and left para-aortic regions.

A possibility of neoplastic etiology likely a neuro-endocrine tumor of pancreatic origin with local and lymph nodal spread was initially considered. However, serum Chromogranin A was not elevated. CECT Chest was done as part of further evaluation, which showed mediastinal and right hilar lymphadenopathy with necrosis. With no clear diagnosis, contrast enhanced MRI (CEMRI) of abdomen was done which showed multiple enlarged periportal, peripancreatic, and retroperitoneal masses with heterogenous enhancement, suggestive of neoplastic etiology likely neuro-endocrine tumor. Patient was referred to the Oncology Department for further work-up, where she was advised for Ga-68 DOTANOC PET/CT imaging and repeat serum Chromogranin A. DOTANOC scan revealed low-grade DOTANOC uptake in pancreatico-duodenal groove, supraclavicular nodes, chest nodes, and right ovary. Repeat Chromogranin A was also not elevated.

Finally, when imaging modalities and laboratory investigation failed to achieve a definitive diagnosis, an ultrasound-guided biopsy was done from the pancreatico-duodenal groove mass lesion. Histopathological examination (HPE) of biopsy specimen showed features suggestive of necrotizing granulomatous inflammation. Tuberculin skin test (TST) was done, and it showed 16 mm induration, conveying a positive test. Patient was started on anti-tubercular therapy (ATT) as a case of pancreatic TB for an initial period of six months and further extended to another 3 months. She responded well to treatment showing both clinical and radiological resolution. Also, her insulin requirement had gradually reduced and stopped. Presently, she is not on any anti-diabetic medication.

### 2.2. Case 2

A 38-year-old lady who was diagnosed to have type 2 diabetes mellitus 6 months ago, which was poorly responsive to oral anti-diabetics (OADs), was started on Insulin 3 months prior. She complains of significant unintentional weight loss of 9 kg over the past 6 months and her present weight is 46 kg. She admits to having had mild, persistent abdominal pain over the same period of time. There was no fever, jaundice, or diarrhoea.

Considering her rapid worsening of glycemic status, lean body mass, and age of onset of diabetes, she was evaluated for possible secondary causes of diabetes. On evaluation, she was hemodynamically stable; general and systemic examination were unremarkable. Her FPG was 226 mg/dL; HbA1C 9.6%, on insulin therapy. On investigation, CBC, liver function tests (Bilirubin, AST, and ALT), and renal function tests (urea and creatinine) were within normal limits. Her thyroid function tests (Free T3, Free T4, and ultrasensitive TSH) were found to be within normal limits. Screening for HIV was negative. Serum electrolytes (sodium, potassium, and calcium) and parathormone levels were within normal range.

USG showed a well-defined hypoechoic lesion measuring 36 × 49 mm of the pancreatic head with minimal vascularity and another 15 × 21 mm sized hypoechoic lesion in peripancreatic lymph node; few retroperitoneal and mesenteric lymph nodes were also visualized.

**Figure 3.**
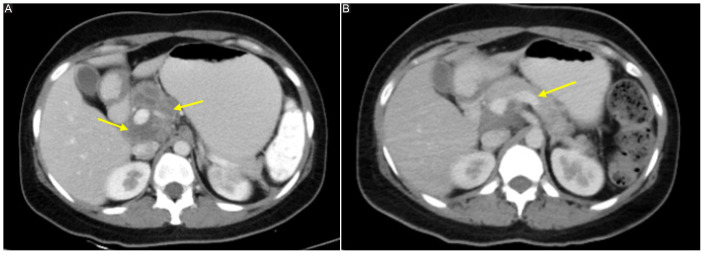
(A) CECT showing pancreatic mass over head of pancreas and adjoining lymph nodes. (B) CECT showing resolution of mass lesion over head of pancreas when compared to initial scan.

CECT Chest, Abdomen, and Pelvis were done which revealed homogeneously enhancing lesion in the region of head of pancreas measuring 48 × 49 × 56 mm in size, also involving the adjacent lymph nodes (Figure 3A). Lesion cannot be separated from the surrounding peripancreatic lymph nodes. Few discrete and conglomerate nodes were seen in preaortic, retrocaval, precaval, left para-aortic, and left inter-aortocaval stations, the largest measuring 27 mm.

CA 19.9, CA 125, carcino embryonic antigen (CEA), alpha feto protein (AFP), and beta human chorionic gonadotropin (HCG) were not elevated and were within normal range. Serum Chromogranin A was also not elevated.

However, endoscopic ultrasound (EUS)-guided fine needle aspiration cytology (FNAC) of peripancreatic lymph node was suspicious of malignancy favoring neuroendocrine tumor. Also, EUS guided fine needle aspiration (EUS-FNA) for GeneXpert turned negative. Hence, Ga-68 DOTANOC PET/CT imaging was done which showed multiple DOTANOC avid lesions over right paratracheal, perivascular, and bilateral hilar lymph nodes. A large coalescent mildly DOTANOC avid centrally hypodense lesion was noted in relation to head of pancreas.

When all imaging modalities, EUS-guided FNAC of peripancreatic lesion and tumor markers yielded ambiguous results; diagnostic laparoscopy with biopsy from pancreatic mass was done. HPE of the biopsy specimen from pancreatic mass revealed necrotizing granulomatous inflammation. TST was positive showing 18 mm induration.

The patient was started on ATT as per national treatment guidelines. She showed remarkable improvement both clinically and radiologically (Figure 3B). Patient started gaining weight and felt better after ATT initiation. She was on ATT for a total duration of 12 months. Her requirement of insulin gradually decreased, and presently, she is off insulin therapy and only on minimal OADs for control of her blood sugar levels.

The demographic, clinical features, investigation, and treatment of the above two cases of pancreatic TB are summarized in Table 1. In both cases, the treating team was initially considering the diagnosis of malignancy. Hence, attempts were made to establish the diagnosis of malignancy rather doing investigation to rule in TB. Hence, EUS-FNA for GeneXpert was not thought and done in the first case. In the second case, ideally, the biopsy should have been taken from the lymph node if malignancy is suspected. However, owing to poor EUS-guided FNAC yield from the peripancreatic node and Chromogranin A repeatedly being normal, it was decided to take biopsy from the pancreatic mass.

### 2.3. Diagnosis

Patients with pancreatic TB frequently undergo extensive investigation which often includes diagnostic laparotomy.^3-5^ In one of the largest and most recent systematic reviews on pancreatic TB by Nikola Panic et al, of the 166 identified pancreatic TB cases, 55.2% of patients underwent laparotomy.^4^ One of the main reasons for subjecting the patient to extensive evaluation is that clinicians often consider pancreatic carcinoma as the provisional diagnosis upfront and hence the diagnosis of pancreatic TB is neglected or not thought of. In both cases, the treating physicians were not considering pancreatic TB as a potential cause of uncontrolled diabetes associated with weight loss and pancreatic mass. Unfortunately, there are no telltale features for pancreatic TB in imaging that could clinch the diagnosis of pancreatic TB, and often the features mimic pancreatic carcinoma.^3,5^ Though the presence of vascular invasion in imaging points toward a diagnosis of pancreatic carcinoma, its presence cannot exclude pancreatic TB.^6^ The mass-forming pattern of pancreatic TB is the commonest form and seldom other uncommon forms like diffuse form and nodular form can also be noted in the imaging of pancreatic TB.^7^

Plasma Chromogranin A estimated through ELISA method has 85% sensitivity and 85% specificity in identifying neuroendocrine tumors making it a reliable, non-invasive modality in detecting neuroendocrine tumors.^8^ Some common clinical conditions leading to false-positive elevation in Chromogranin A include use of proton pump inhibitors, presence of impaired kidney function, atrophic gastritis, heart failure and strenuous exercise before the test.^9^ However, the absence of elevated tumor markers can be counted against pancreatic carcinoma.^10^ In both patients, neuroendocrine tumor was thought to be the diagnosis at initial stages based on imaging findings though serum Chromogranin A was not elevated on repeated occasions.

Hence, the patients were subjected to DOTANOC scan. DOTANOC scan has revolutionized the traditional imaging approach in neuroendocrine tumors. It is recommended as the primary imaging investigation for localization, tumor characterization, staging and re-staging, and monitoring of patients with neuroendocrine tumors.^11^ Ironically, the second patient had increased DOTANOC uptake, suggesting that even DOTANOC scan may be non-specific in differentiating pancreatic TB from malignancy (neuroendocrine tumor). It is not uncommon to get false-positive results in DOTANOC scan. Lymphoid hyperplasia leads to false-positive DOTANAOC uptake, and this may be the reason for increased DOTANOC uptake in the second case. In a study by Yusuf Menda et al, 8% of the Ga-DOTANOC PET/CT scans showed false-positive results leading to unnecessary small bowel resection.^12^

The cornerstone to the diagnosis of pancreatic TB is through obtaining appropriate specimens for microbiological and histopathological analysis.^13^ Studies on pancreatic TB suggest that EUS-FNA gives a good yield for diagnosing pancreatic TB.^14^ EUS-FNA is more accurate and superior to conventional imaging like CT and MRI in detecting pancreatic lesions. It not only avoids surgical procedures which are not warranted but also is the preferred intervention for loco-regional staging of pancreatic carcinoma.^15^ Since the procedure is invasive though minimally, conventional imaging and tumor markers are usually performed prior to it. EUS-FNA is used for diagnosing the suspected mass lesion of pancreas and its negative predictive value is low.^15^ Though the procedure is of high utility value, certain pitfalls like requirement of technical expertise which involves a lengthy learning period and availability of cytopathologist precludes its use in resource-limited settings.^14,15^

In both cases, the diagnosis was made by the HPE of the pancreatic mass biopsy. In the second case, FNAC of the peripancreatic lymph node was suggestive of malignancy; however, biopsy from the pancreatic mass showed necrotizing granulomatous inflammation, suggesting pancreatic TB. This concludes that FNAC of lymph nodes may be non-specific, and HPE of the pancreatic mass is superior to FNAC of the lymph node in diagnosing pancreatic TB.

TST is often positive but a non-specific investigation for pancreatic TB especially in high-endemic regions for TB. However, in certain conditions where the diagnosis of pancreatic TB and carcinoma is contemplated, TST may point toward a diagnosis of TB. TST is of immense diagnostic value in detecting pancreatic TB where there is always a diagnostic dilemma between TB and cancer. The easy availability, low cost, non-requirement of technical expertise in performing the test, early interpretation of results, and reliability make TST the preferred investigation at the initial stages. Present guidelines recommend that where facility exists, TST or interferon gamma assay to be performed at the initial stages on suspecting pancreatic TB.^13^ Considering the utility of EUS-FNA and TST, it is suggested that TST may be done at the initially stages for all suspected cases of pancreatic TB, followed by EUS-FNA, preferably from pancreatic mass, prior to other invasive procedures like laparotomy. Unfortunately, despite being an easily available test, TST is not routinely done upfront in pancreatic mass lesions, and patients frequently end up in invasive investigations. The authors strongly encourage the routine use of TST as the initial investigation of choice in all pancreatic mass lesions, prior to any high-end radiological and invasive procedures, in line with present guidelines.

### 2.4. Treatment

Pancreatic TB responds well to standard anti-tubercular therapy (ATT), seldom requiring intervention in the form of surgery or fluid drainage.^16^ The training module on extra-pulmonary TB, 2023, spelt out by the Ministry of Health and Family Welfare, Government of India, has encompassed the treatment of pancreatic TB under management of abdominal TB.^1^ Standard 04 drug ATT, which includes isoniazid (H), rifampicin (R), pyrazinamide (Z), and ethambutol (E), is given for the initial 2 months followed by 3 drug ATT (HRE) for the next 4 months. The decision to extend the treatment beyond 6 months is taken by the treating physician on a case-to-case basis. Systematic review by Panic et al also showed similar trend in the treatment of pancreatic TB where almost all the patients required ATT (98.2%) and only 24.1% required surgical intervention.^4^

In both cases, ATT as per guidelines was started, and both patients showed clinical and radiological resolution. Also, both patients showed remission in diabetes. The first patient did not require any anti-diabetic medication including OADs after 6 months of ATT, while the second patient is on minimal OADs, off insulin therapy for control of blood sugar. Surgical intervention was not required to treat in either of the patients.

## 3. Discussion

The global burden of TB especially in developing countries is well known and needs no special emphasis. While lungs (pulmonary TB) remain the most common affected organ in TB, extra-pulmonary tuberculosis (EPTB) constitutes 16% of the total TB cases.^1^ Pancreatic TB, a form of EPTB, is considered very rare, and there are only a handful number of case series. While 87% of the total globally notified TB cases are from 30 high-burden countries mostly from Asia, a systematic review of 166 pancreatic TB cases by Nikola Panic et al suggests that about 45% of pancreatic TB are from developed countries of North America, Australia, New Zealand, and Europe.^1,4^ The skewed demographic data suggests that the identified cases of pancreatic TB in developing counties are just the tip of iceberg and many cases either remain undetected or wrongly diagnosed.

2 cases of pancreatic TB from 2 different tertiary care hospitals, identified almost at the same period, are discussed here. The treating physicians were not aware of the other case that was identified in a different center while evaluating the patient. The experience of identifying and treating the pancreatic TB cases were shared and collated later. Both the cases of pancreatic TB described are young females. The mean age of 166 patients described by Nikola Panic et al is 41.61 ± 13.95 years.^4^ Cases reported from India suggest that more than 50% of pancreatic TB patients are <30 years old.^17-19^ In contrast, pancreatic cancer, which closely mimics pancreatic TB, is a disease of the elderly and is seldom seen before 55 years of age.^20,21^ There is no clear predilection of pancreatic TB to any gender though many studies report pancreatic TB to be more common in men.^5^ In both cases, there was no previous history of TB, similar to other studies where only 7.3% of pancreatic TB patients had previous history of TB.^4^

### 3.1. Clinical characteristics

Both the patients presented with significant unintentional weight loss and diabetes mellitus with uncontrolled blood glucose levels. Both the patients admitted to having abdominal pain in the period prior to presenting to medical OPD, though it was not the presenting symptom. Also, both the patients did not have fever, jaundice, or diarrhoea. However, studies on pancreatic TB including systematic reviews suggest that fever and abdominal pain are the most common symptoms, followed by the history of weight loss.^4,20^ Jaundice and diarrhoea were the presenting symptoms in less than 20% of the patients.^3,4,20^ The relationship between TB and diabetes mellitus is two way and well established. Studies have shown that while individuals with diabetes mellitus are at higher risk of acquiring TB, pancreatic TB can cause secondary diabetes through destruction of islet cells in pancreas.^22-24^ Though diabetes mellitus secondary to pancreatic TB is documented, poor glycemic status or uncontrolled diabetes mellitus is rarely described as the presenting symptom of pancreatic TB in the available literature. Both our patients had weight loss associated with poor glycemic control on presenting to the medical OPD. This article emphasizes the need to look for secondary causes of young onset or rapidly worsening diabetes mellitus with pancreatic TB as one of the potential causes.

Pancreatic mass was frequently seen in pancreatic TB, being the most common presenting symptom seen in 80% of the cases.^4^ The most common site of pancreatic mass is over the head of pancreas, seen in both the patients as well.^3,4,20^ Pancreatic TB may present with dyspeptic symptoms, although they are not specific.^25^ Pancreatic TB rarely presents with acute/chronic pancreatitis.^26^ Peripancreatic lymph nodes are frequently involved, seen in approx. 50% of patients with pancreatic TB.^4^ Although both the patients were HIV negative, a resurgence in abdominal TB in western countries due to HIV is reported, and hence screening for HIV is mandatory in the work-up to pancreatic TB.^27^

### 3.2. Critical review

The diagnosis of pancreatic TB remains elusive as there are no pathognomonic clinical or radiological features. Due to the rarity of the disease, pancreatic malignancy is often considered as the diagnosis by the treating physician. The availability of cytopathologists and facilities for EUS-FNA is not easily available in majority of the health setups in many countries. Low-cost, easily available, but highly useful investigation like ESR and tumor markers (Chromogranin A, CA 19.9) need special emphasis in the treatment guidelines. None the less, the key to the diagnosis of pancreatic TB is early suspicion.

There is no clarity on the ideal site for FNAC. FNAC from the mass lesion may predispose to seeding of the cancer cells if it is malignancy, while FNAC from the adjoining lymph nodes or peripancreatic lesions may still not identify pancreatic TB.

## 4. Conclusion

Pancreatic TB closely mimics pancreatic cancer. Hence, it is essential to suspect pancreatic TB in an appropriate clinical setting. While pancreatic TB is commonly seen in younger age groups, pancreatic carcinoma is usually a disease of the elderly. Pancreatic TB frequently presents with fever and abdominal pain. Significant unintentional weight loss and diabetes in young should prompt physicians in suspecting pancreatic TB. There are no telltale features for pancreatic TB in imaging that could clinch the diagnosis of pancreatic TB, and often the features mimic pancreatic carcinoma. The cornerstone to the diagnosis of pancreatic TB is obtaining an appropriate specimens for microbiological and histopathological analysis. TST may be done at initial stages for all suspected cases of pancreatic TB, followed by EUS-FNA, preferably from pancreatic mass, prior to other invasive procedures like laparotomy. Pancreatic TB responds well to standard ATT, seldom requiring intervention in the form of surgery or fluid drainage. ATT initiation leads to remission of diabetes secondary to pancreatic TB. The authors recommend considering the diagnosis of pancreatic TB in all cases of pancreatic mass lesions particularly in young individuals. ESR and tumor markers need to be done upfront before any invasive investigation. Where there is a diagnostic dilemma between TB and malignancy, EUS-FNA should be done. Formulating a management protocol by national agencies suiting their needs, considering the medical resources available, is of paramount importance.

## Conflict of Interest Statement

The authors have no financial or other conflicts of interest to disclose.

## Consent and Ethical Clearance

Written consent was obtained from both the patients described in our study. Also, ethical clearance was obtained from the institutional ethics committee, vide IEC/04/2023/35 dated 06 Feb 23.

## Author Contribution Statement

All authors contributed either in all or part of the study conception, management of the patients, data collection, preparation and editing of the manuscript, literature review, validation, and interpretation of results. All authors reviewed the results and approved the final version of the manuscript.

## Figures and Tables

**Table 1.** Demographic, clinical features, investigation, and treatment of two cases of pancreatic tuberculosis.

	**Patient 1**	**Patient 2**
Age (years)	26	38
Gender	Female	Female
Co-morbidities	Nil	Diabetes, 6 months
Previous history of tuberculosis	Not present	Not present
Presenting symptom	Significant unintentional weight loss of 2 months duration	Significant unintentional weight loss of 6 months dura tion
Duration of symptoms	3 months	6 months
Blood glucose on presentation	427 mg/dL	226 mg/dL
HbA1C on presentation	10.1%	9.6%
HIV screening	Negative	Negative
Pancreatic mass	Present	Present
Location of pancreatic mass	Head of pancreas	Head of pancreas
Extrapancreatic involvement	Present, Peripancreatic lymph nodes	Present, Peripancreatic lymph nodes
Initial presumptive diagnosis	Malignancy (neuroendocrine tumor)	Malignancy (neuroendocrine tumor)
Chromogranin A (done twice)	Not raised	Not raised
Tumor markers CA 19.9, CA 125, CEA, AFP, βHCG	Not done	All negative
Ga-68 DOTANOC PET/CT scan	Low-grade DOTANOC uptake	Multiple DOTANOC avid lesions
FNAC of lymph node	Not done	Suspicious of malignancy favoring Neuroendocrine tumor
EUS FNA GeneXpert	Not done	Negative
HPE of biopsy (pancreatic mass)	Necrotizing granulomatous inflammation	Necrotizing granulomatous inflammation
Treatment	Anti-tubercular drugs	Anti-tubercular drugs
Treatment duration	9 months	12 months
Response to therapy	Both clinical and radiological resolution, remission of diabetes	Both clinical and radiological resolution, remission of diabetes
Complications	Nil	Nil
